# A prospective cohort study of game-based learning by digital simulation of a pig farm to train agriculture students to reduce piglet mortality

**DOI:** 10.1186/s40813-018-0105-6

**Published:** 2018-12-01

**Authors:** Karl Johan Møller Klit, Ken Steen Pedersen, Helle Stege

**Affiliations:** 1 University of Copenhagen, Faculty of Health and Medical Sciences, Department of Veterinary and Animal Sciences, HERD, Grønnegårdsvej 2, 1870 Frederiksberg C, Denmark; 2 Ø-Vet A/S, Køberupvej 33, 4700 Næstved, Denmark

**Keywords:** Agriculture, Animal welfare, Digital simulation, Game-based learning, Piglet mortality

## Abstract

**Background:**

Acquisition of knowledge and skills by agriculture students prior to real-life experience is a well-known educational challenge. Game-based learning has the advantage of being active, experiential, and problem-based, and provides immediate feedback. Simulation games are widely used in other fields to support traditional teaching methodology and actively engage students. This study investigates whether a digital pig farm game can assist agriculture students in acquiring knowledge and skills in farrowing management to reduce mortality in piglets prior to weaning.

**Results:**

Overall the simulation group tended to score higher; however, at 5% confidence level, the difference was not significant. The simulation group had the lowest standard deviation which to some extent was due to reduced number of low-scoring students. Nevertheless, students requested more digital simulation for learning and practicing skills.

**Conclusion:**

The use of game-based learning in agricultural education has a huge potential for building skills needed on a real pig farm. However, an increase in knowledge related to farrowing management was not documented.

The integration of game-based learning into an educational setting needs further evaluation.

## Background

With the use of highly prolific sows in swine production, high piglet mortality before weaning remains an unsolved problem. The annual report from selected EU countries shows that preweaning mortality rate increased from 12.9% in 2014 to 13.3% in 2015 [1]. Hypothermia, starvation, and crushing are the primary causes of mortality in piglets during the first 48 h after birth [2, 3]. Therefore, improved management skills are crucial to reduce the mortality rate [4]. Despite general agreement that these skills are vital, there is little opportunity for students to practice them before being employed on a pig farm. At agricultural colleges novice students are presented to skills they must master when working on pig farms, but training is when they are employed on farms. The training of international labour is primarily the responsibility of the individual farmer. International labour has often no experience from pig production and start from zero when they are employed in Denmark. However, it is mandatory that all employees who are going to inject livestock animals must complete an 8-h medication course. To achieve better training of new international employees and to offer Danish agricultural schools new learning tools, we have developed “Game of Piglets.” The game is a virtual pig farm that has as its objective to allow students to practice procedures involved in external biosecurity and farrowing management. “Game of Piglets” belongs to the genre of simulation/ adventure games. Core competencies highlighted in the game are farrowing aid, recognition of sick sows after farrowing, aseptic conditions in surgical interventions, and optimal piglet environment. These competencies are developed by a series of tasks that must be solved in a particular order, just as in a real pig herd. The game’s scoring system is a piglet survival barometer. To see how the task of farrowing assistance are created please follow the link: https://www.youtube.com/watch?v=ukmxfhVT-zQ

**Fig. 1:**
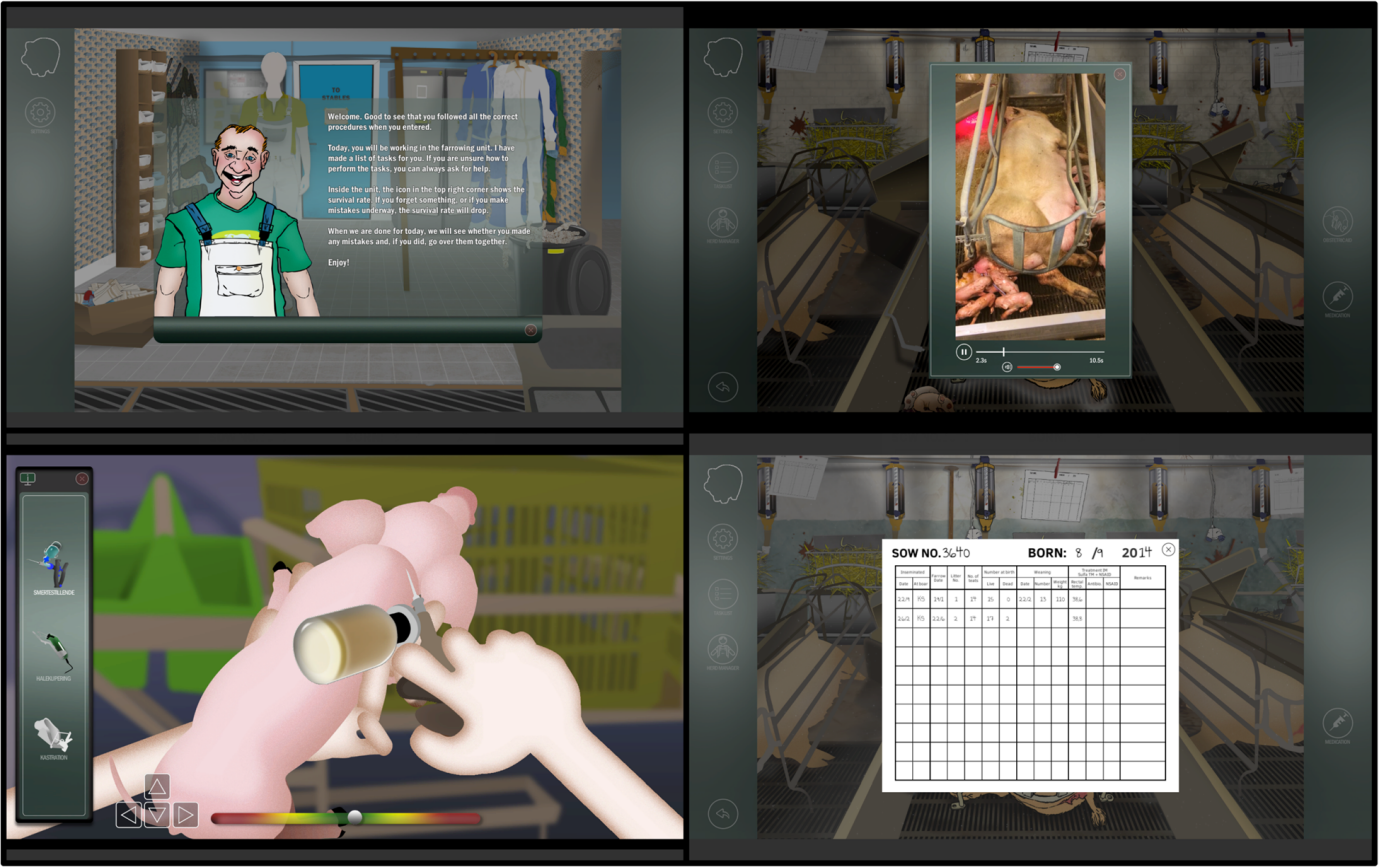
Screenshots from “Game of Piglets”

If a task is performed incorrectly or omitted, it results in decreased piglet survival. At any time in the game, students have the opportunity to ask the farm manager about how to solve the tasks. When all tasks are completed and the student leaves the farm, an evaluation of tasks resolved correctly and/or a description of the mistakes that were made will be given. Screenshots from the game is displayed in Fig. 1.

**Fig. 2:**
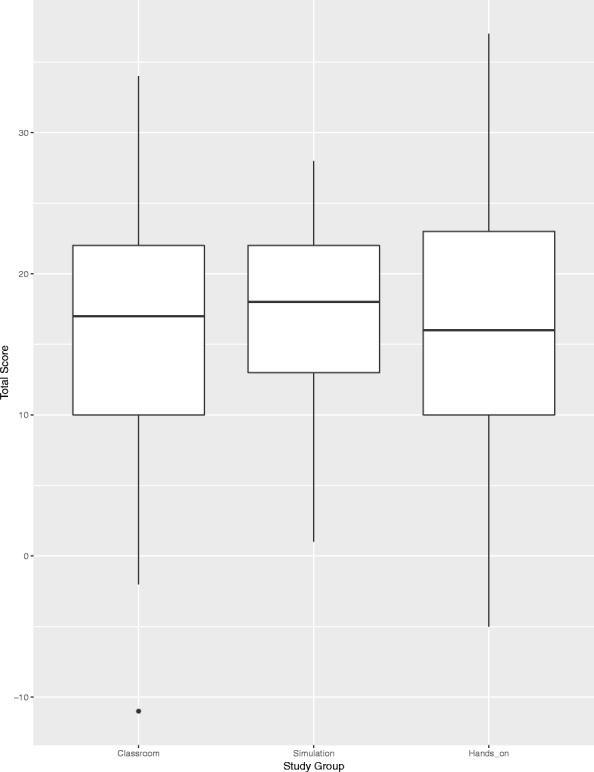
Box plot of the multiple-choice test score for each group. Multiple-choice test consisted of 20 questions. An incorrect answer gave minus 1 point, no answer 0 points and correct answer 2 points. Points from each question were summarised to a total score from the test. The difference found between the groups was not significant

Learning games are widely used in other disciplines such as health, business, military, and mathematics [5]. The most popular learning game genre by far is simulation, but other genres as action, adventure, strategy, and puzzle are also used [5]. Simulation training in health profession educations is associated with positive effects on knowledge acquisition, skill, and behaviour [6]. Virtual patients are extensively used for skills training in undergraduate nursing educations [7]. Virtual patients allow nurses to learn and practice in a safe, virtual environment where time can be paused. The pause allows time for self-analysis [8]. Thus far learning games for veterinary use is increasing, but only a few learning games have been developed specifically for agriculture education. In this paper we will introduce a learning game for agriculture students designed to help them improve their theoretical and practical skills in the field. The objective of the study is to measure knowledge acquisition in farrowing management among students only exposed to classroom instruction, students whose education was supplemented by “Game of Piglets,” and students who also had hands-on instruction. Furthermore, students’ perceived self-efficacy surrounding the practical skills of farrowing management was also measured.

## Methods

### Study design

A three-arm cohort study with negative and positive control groups was conducted at four Danish Agriculture colleges. Requirements for conducting the study were to conflict minimal with the curriculum at the schools and implement the study into the schedule. Small changes in the schedule were necessary to ensure teaching in farrowing management at the start of the course. Students were divided into three groups at each school. Each group received instruction covering farrowing management but with three different didactic approaches. All groups received traditional classroom instruction. To ensure that classroom instruction had the same content between schools, a list of learning goals were shared between responsible teachers. These learning goals were all about management around farrowing. The subject farrowing management was for all schools integrated in a larger course about general husbandry. Farrowing management was taught in classroom between two lectures (90 min) to four lectures (180 min) between schools. Group one (classroom, negative control) did not receive any further instruction. Group two (simulation, exposure group) used virtual simulation of farrowing management for two hours. Group three (hands-on, positive control) had a half-day expedition to a farm with hands-on instruction. The farm visits had the objective to introduce students to see and try tasks in a farrowing unit. Teachers encourage students to train the skills on the visit, but this was not mandatory for the students. One week after exposure, students were given a multiple-choice test and a self-efficacy questionnaire to test the hypothesis. To measure knowledge acquisition in farrowing management amongst the groups, a multiple-choice test was conducted. The book “Developing and Validating Multiple-choice Items” [9] was used to construct the test. The test was a 20-question multiple-choice test with three possible answers per question. Two full-time swine veterinarians were chosen to peer-review the questions. Furthermore, 16 students from the Agriculture College with experience from swine production validated the test. To reduce guesswork, the scoring in the test awarded 2 points for a correct answer, − 1 points for an incorrect answer and 0 points for no answer. The total score from all 20 questions was summarized in a final score as a continuous variable. If the respondent had given two answers for one question it was registered as incorrect. A questionnaire was constructed to measure students’ perceived self-efficacy. Perceived self-efficacy evaluation is a method to measure how students judge their own competencies. The methodology relies on the theory of Albert Bandura. To summarize, he claims that there is an association between the level of perceived self-efficacy and the performance of a certain skill [10]. Students were given statements about specific skills in farrowing management. They were asked to rate each statement on a scale from zero to seven. Zero was “cannot do at all” and seven was “highly confident can do.” Finally, the simulation group completed a satisfaction survey after the gaming session.

### Setting

Danish agriculture colleges (17 in Denmark) are independent private institutions. Most of the Danish agriculture colleges (13 represented) are organized under “Danske Landbrugsskoler” [11]. This organization takes care of the educational policy interests of agricultural schools, and strengthens networking and cooperation between schools in the educational, academic, and institutional areas. However, teachers in swine husbandry are also organized under the so-called “Hyo-academy.” The Danish Agriculture & Food Council [12] facilitates this academy with 17 agricultural colleges represented. Four schools from the “Hyo-academy” volunteered to participate in this study. The study was conducted from January 2017 to March 2017. During this period, new students began the 20 weeks “Basic Course Two” in general husbandry at all colleges. All schools follow the same program over the year. For the majority of students, this was their first experience with farming animals and agriculture in general.

### Sampling

Multiple choice test scores were used to calculate the sample size. The difference in means was set to two points and the standard deviation was set to four. Using a confidence level of 95% and power of 80% the necessary size of each group was calculated to 63 students, giving a total of 189 students in the study. The inclusion criterion for the colleges was a minimum of 21 students enrolled in the class. The college should also be able to facilitate partitioning of students into three groups throughout the study period. Partitioning was done with respect to the existing curriculum, in collaboration with the course coordinator from each school, to maximize randomization of the groups. In two of the schools, students were partitioned into three independent classes. In the two other schools, the students were partitioned within the class. Each college was requested to facilitate classroom instruction and hands-on instruction. The investigator administered the virtual simulation experience at all schools. One week after exposure, all students were tested with the multiple-choice test and the self-efficacy questionnaire. All answers were obtained with all students gathered in one room. Oral instruction was given in how to fill out the questionnaire. A written description was also provided. The questionnaire was administered in paper forms. Students returned the questionnaire after answering and left the room. The questionnaire was constructed in three modules. The first module determined demographical information by means of closed questions: gender, age, experience in swine production, and gaming behaviour. Finally, students were asked in an open question to state their favourite digital game. The second module was the multiple-choice test, and the third module was the perceived self-efficacy evaluation.

### Statistical analysis

The data was anonymized and entered into Microsoft® Excel® for Mac 2011 and exported into statistical software R-studio version 1.1.383. A descriptive analysis was initially performed using different plot and summary statistics. The summarized average test score from the multiple-choice test was tested for statistical differences between study-groups using a univariable ANOVA-test. Differences between standard deviation (SD) were tested using an F test. Next, a multivariable model was constructed with the outcome test score and tested against all predictive variables (school, study group, age, sex, experience, background, play behaviour, farm simulator). Backwards elimination was performed using Akaike’s Information Criterion (AIC) test. Each skill in the perceived self-efficacy questionnaire was tested for statistical difference between study groups using the Kruskal-Wallis test. This test has the limitation of being unable to distinguish between groups that differ. Therefore, if statistical differences were found, the Wilcoxon signed-rank test was used to identify which groups differed from others. If respondents had marked two numbers in the self-efficacy questionnaire, the score was rounded down to the closest whole number. The level of significance was set to 95% in all analyses.

## Results

Usable multiple-choice test and perceived self-efficacy questionnaire results were obtained from 186 students. Students from the simulation group 49 out of 62 also responded to a satisfaction survey after the gaming session. One respondent was excluded because it was impossible to determine which group the respondent belonged to. In total, the gender distribution was 159 males (85.5%) and 27 females (14.5%). The average age was 17.2 years and the median was 17. Forty-four students (23.7%) had experience in pig production and 142 (76.3) had no experience. In the open question about favourite digital game, 52.3% answered “Farming Simulator 2017”. The demographical information of the students can be seen in Table 1.

**Table 1:** Demographical information of students

Total students (*n* = 186)	Classroom 67 (36%)	Simulation 62 (33.3%)	Hands-on 57 (30.7%)
Mean age (median)	17.4 (17)	17.2 (17)	17.0 (17)
Gender			
Male	60 (89.5%)	52 (83.9%)	47 (82.5%)
Female	7 (10.5%)	10 (16.1%)	10 (17.5%)
Experience^a^			
Yes	19 (28.4%)	12 (19.4%)	13 (22.8%)
No	48 (71.6%)	50 (80.6%)	44 (77.2%)
School			
1	26 (38.8%)	16 (30.8%)	18 (31.6%)
2	15 (22.4%)	25 (40.3%)	20 (35.1%)
3	17 (25.4%)	14 (22.6%)	13 (22.8%)
4	9 (13.4%)	7 (11.3%)	6 (10.5%)
Background^b^			
Basic course one	26 (38.8%)	44 (71%)	23 (40.4%)
Employed on a farm with agreement from school	24 (35.8%)	12 (19.4%)	21 (36.8%)
Employed on a farm with no agreement from school	11 (16.4%)	2 (3.2%)	8 (14%)
Other	6 (9%)	4 (6.4%)	5 (8.8%)
Gaming behaviour			
Never	8 (11.9%)	7 (11.3%)	5 (8.8%)
Once a month	8 (11.9%)	8 (12.9%)	7 (12.3%)
Once a week	11 (16.4%)	10 (16.1%)	5 (8.8%)
Several times per week	25 (37.3%)	20 (32.3%)	17 (29.8%)
Once every day	3 (4.5%)	6 (9.7%)	10 (17.5%)
Several times per day	11 (16.4%)	11 (17.7%)	13 (22.8%)
Farming simulator as favourite game			
Yes	30 (44.8%)	36 (58.1%)	32 (56.1%)
No	37 (55.2%)	26 (41.9%)	25 (43.9%)

^a^Experienced students had earlier a paid job on a pig farm or born on a pig farm

^b^Background: Employment on a farm is not only pig farms

**Table 2:** Multiple-choice test values for students exposed to three different didactic approaches

Exposure	*n*	Mean*	SD*
Classroom	67	15.9^a^	8.31^a + b^
Classroom + Simulation	62	17.4^a^	6.76^a^
Classroom + Hands-on	57	16.4^a^	9.35^b^

*Means and SD with different superscripts are statistically significantly different (*P* < 0.05)

The summarised mean score from the multiple-choice test is displayed in Table 2 and the box plot in Fig. 2. The simulation group had the lowest SD at 6.76. A significant difference between the simulation group and the hands-on group was found at 28% (*P* = 0.01). Between the simulation group and the classroom group we found a difference of 19% (*P* = 0.1), but not significant.

On average, the simulation group had slightly higher scores than the negative control group (difference = 8.4%) and the hands-on group (5.6%). In addition, there was a significantly smaller standard deviation among students in the simulation group compared to the hands-on group, which to some extent was caused by a lower number of students with wrong answers. However, the differences in SD were not significant between the simulation and classroom groups.

**Fig. 3:**
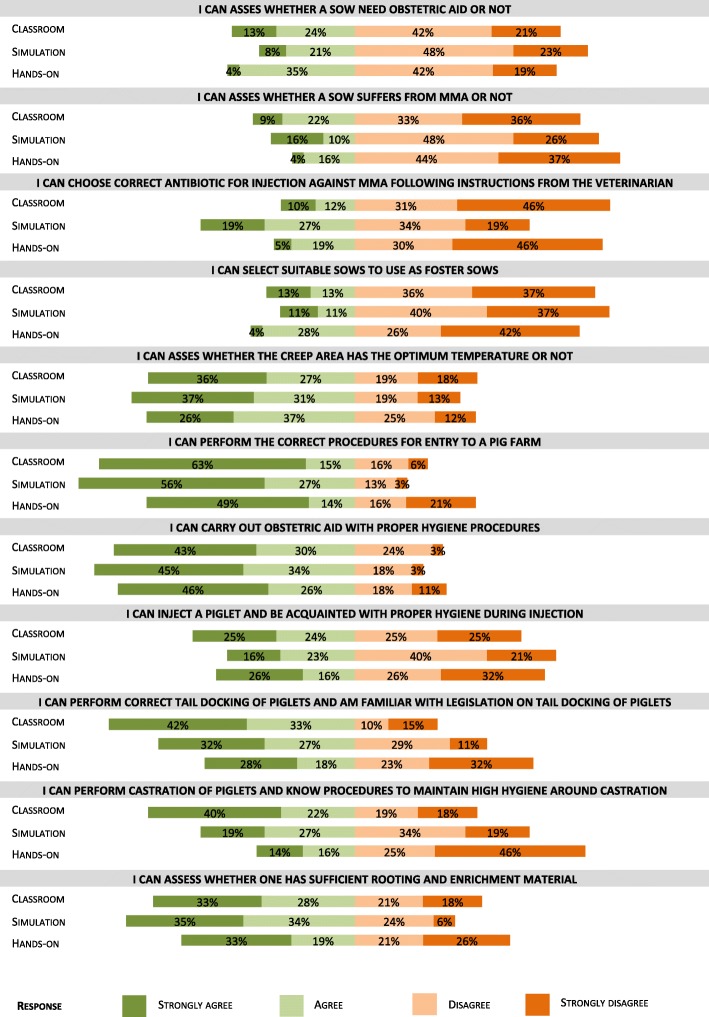
Perceived self-efficacy responses to statements about farrowing management and external biosecurity. Self-evaluation of competencies in relation to 11 statements as referred to as farrowing management and external biosecurity. The simulation group has evaluated significantly higher when it comes to choosing the correct antibiotic against MMA. The hands-on group has evaluated significantly lower in the conditions of tail docking and castration

For the self-efficacy questionnaire, the distribution of answers is displayed in the diverged stacked bar charts in Fig. 3.

**Fig. 4:**
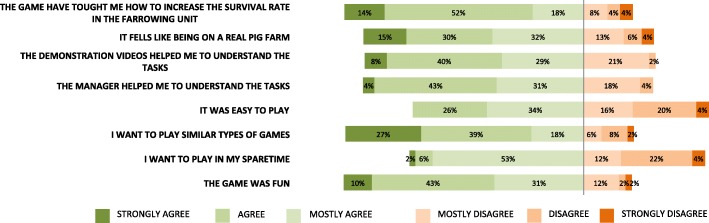
Responses to satisfaction questionnaire about game session experience. Simulation group (*n* = 62) filled out a satisfaction survey after gaming session. Overall the responses were positive

In response to the statement “I can choose the correct antibiotic for injections against MMA following instructions from the veterinarian,” the simulation group scored significantly higher (*P*-value 0.001) than both the classroom and hands-on groups. For the statement “I can perform the correct procedures for entry to a pig farm”, the hands-on group scored significantly lower than both the classroom and simulation (*P*-value 0.04) groups. In responding to the statements dealing with tail docking and castration, the hands-on group scored significantly lower than both the classroom and simulation groups (P-value 0.03 and *P* < 0.001, respectively). Answers to the satisfaction survey are displayed in Fig. 4.

After playing the simulation game, 84% of the students agreed that they had learned how to increase the survival rate of piglets in the farrowing unit. 84% also agreed that they enjoyed playing and wanted to play other learning games similar to “Game of Piglets.” However, 38% answered that they did not want to play this game in their spare time, and 40% found it difficult to play “Game of Piglets.” The difficulties were due to navigation and the lack of immediate feedback in the game. The lack of feedback was a deliberate design choice to encourage cognitive thinking rather than trial and error.

## Discussion

The present results indicate that students in Danish agriculture schools enjoy using digital learning games. The results of the multiple choice test were not significant, but indicated that using a simulation game in the curriculum had a positive effect on learning outcome and improved results in particular in the lower-performing half of students. It was anticipated that both the simulation and hands-on groups would perform significantly better than the classroom group in the multiple choice test. The multiple choice test was chosen to evaluate many students with low amount of resources. The difficulty of the test was carefully designed to assess skills and knowledge about farrowing management. A pre-test before instruction could have verified if students were able to answer correct even without instruction. The exposure time for both the simulation and hands-on groups was set to two hours. Two hours were the time that was planned in the schedule for a farm visit in the course on all schools. Therefore the time for gaming was also set for two hours to unify the groups as much as possible. This is likely too short an exposure time to have a significant effect on memorizing and retaining knowledge of farrowing management. The real farm is not an optimal learning environment if time is sparse, as there are many distractions on a real life pig farm. Therefore, the intended learning objectives may often become secondary on a first-time pig farm visit. The pig farm is more optimized for learning when students are familiar with the smell, sound and the condition in a pig farm. The simulator was easy to install, but it took some time and effort to become familiar with. Most of the students became competent at navigating and using the functions in the simulator during the exposure time. It is established that repetition and experience have a positive effect on memory and learning [13]. From this perspective, more exposure time to either simulation or hands-on experience could have changed the outcome of the study. Additionally, agriculture students claim that they learn much more from practical training than from classroom instruction. This opinion was found through a qualitative study conducted on Danish agriculture colleges in 2016 in which students were asked about the relationship between school and practical training [14]. However, the results of the multiple-choice test did not support that finding. The perceived self-efficacy questionnaire shows that the hands-on group scored significantly lower when it comes to procedures for entering a pig farm as well as for tail docking and castration. It was expected that the hands-on group would score highest since they had experienced reality and thereby have the best prerequisite for doing these procedures. Nevertheless, the low score could be attributed to the fact that these students experienced the complexity of reality. This may have increased their anxiety and reduced perceived self-efficacy because of exposure to reality without proper and thorough preparation. In surgical courses at veterinary schools, students have a high level of anxiety prior to surgical procedures on live animals. But with simulated skills lab training on models beforehand, anxiety levels before performing live surgery decreased significantly [15].

Assessment of the efficacy of educational interventions often includes Kirkpatrick’s Four-Level Model [16]. Level 1 is evaluation-reactions, Level 2 is evaluation-learning, Level 3 is evaluation-transfer, and Level Four is evaluation-results. The students’ positive response (Level 1) to the satisfaction questionnaire indicates that they had a positive experience with “Game of Piglets.” Although these positive reactions are subjective and are not the sole determinant of learning, they play a key role in assessing student engagement that in turn enhances active learning and understanding. Still, such a survey data collection tool has the inherent limitations of subjectivity, that is, self-report bias. Evaluating progress in learning (Level 2) moves beyond learner satisfaction and attempts to assess whether students have advanced in skills and/or knowledge as a direct result of the educational intervention. To this end we developed the multiple-choice test as a tool for measuring knowledge acquisition, and the perceived self-efficacy questionnaire for assessing skills. Despite this, it is still difficult to attribute the improvements in knowledge and skills acquisition solely to the use of tailored educational games. Measuring Levels 3 and 4 is costly and time consuming, as the students’ acquired professional knowledge and skills must be evaluated through demonstration and clinical examination. These are the recognized limitations of this study.

## Conclusion

The results of this study show that custom designed digital educational game simulations of pig herds can be used as an additional training resource for undergraduate agricultural students. Though not significant, the results indicate that the simulation game had a positive effect on learning outcome and tended to improve results, especially among lower-performing students. The students’ commitment to game-based learning was positive and they requested more game modules with which to practice their skills. Digital games offer the new generation of teachers an opportunity to mix traditional approaches with simulation training. More studies are needed to explore the pros and cons of game-based learning in agricultural education.
